# 

**Published:** 1924-04

**Authors:** 


### THE Hospital and Health REVIEW

A JOURNAL FOR EVERYONE INTERESTED IN PUBLIC HEALTH, PREVENTIVE AND CURATIVE MEDICINE AND HOSPITAL AND INSTITUTIONAL WORK.

*Established 1886.*

### Published MONTHLY, Price 6d.

THE HOSPITAL AND HEALTH REVIEW includes within its sphere the various aspects of PUBLIC AND PERSONAL HEALTH, PREVENTIVE AND CURATIVE MEDICINE, HOSPITAL AND INSTITUTION WORK, AND THE GENERAL WELL-BEING OF THE PEOPLE. These subjects of truly vital concern are dealt with in plain, and as far as possible non-technical, language, so that they may be understood, and consequently of *service to everyone* interested in these matters.

Price 6d. of all Booksellers and Newsagents, or 7½d. post free of the Manager, "The Hospital and Health Review," 28/29 Southampton Street, Strand, London, W.C. 2.

### Local Medical Notes.

University of Bristol.—Students of the University have recently passed the following examinations :—

M.B., Ch.B.—(First Class Honours) : J. A. James. (Second Class Honours) : A. P. Bodman, H. Taylor. *Pass :* N. J. Bown, G. B. Bush, W. L. Cossham, F. R. Edbrooke, N. J. England, H. M. Golding, R. C. Hatcher, A. G. Heron, F. G. Jenkins, M. V. Joscelyne, P. C. Joscelyne, E. C. K. Kenderdine, E. F. King, C. H. Osmond, R. A. Sammons, R. E. Whitby, C. F. Wilson. *Final Examination, Part I. :* A. F. Alford, K. F. Alford, D. H. Beatson, E. E. Benson, H. W. Brassington, D. E. Crellin, S. J. H. Griffiths, C. I. Ham, F. H. Hollingshead,

J. A. Hooker, C. T. Hyatt, A. H. Lowther, R. E. Lucas, C. P. Porter, D. C. Prowse, L. G. Robertson, H. Rogers, J. F. Southward, G. M. Terrell, M. M. TeWater, T. W. Ware, K. M. Willmore, G. M. Minifie. *2nd Examination, Part II.:* J. C. Batt, O. J. P. Bollon, J. M. Camps-Campins, A. M. Critchley, T. F. Hewer, R. Jackson, M. Srisvasti, E. R. Wide.

D.P.H.—*Part I.:* E. G. Don.

B.D.S.—E. K. Tratman. *2nd Examination (in Dental Anatomy only):* E. C. Browne.

Diploma in Dental Surgery.—R. G. Downes, G. J. Kelly, B. F. Taylor. *2nd Examination:* S. Phillips. *In Dental Mechanics and Metallurgy:* C. S. Cossham. *1st Examination:* W. H. Greet, E. W. Mogg, C. S. Wilde, C. P. Winstone. *In Dental Anatomy only:* D. M. Cox, R. W. Pinniger. *In Anatomy only:* T. D. Lewis.

L.D.S., R.C.S. Eng.—J. W. Cooper, C. E. Downe.

The **Royal Medical Benevolent Fund** is appealing for increased support. The Fund assists, by grants and small annuities, medical men and their dependents who through ill-health or misfortune are in great poverty. During the last two years very heavy calls have been made on the resources; the help given has in many cases necessarily been less than adequate. The charity is non-contributary, and depends on the generosity of members of the profession. We commend to each one the claims of this very deserving effort to help the less fortunate brethren. Subscriptions should be sent to the Local Honorary Secretary, Mr. E. Watson-Williams, 12 Victoria Square, Clifton, Bristol; or to the Treasurer, 11 Chandos Street, London, W. 1, either of whom will be pleased to give any further information.

**British Medical Association Prize.**—The Medical Faculty of our University is to be congratulated on the success of Miss B. Y. de la Pasture in being awarded one of the prizes offered by the British Medical Association to medical students of the United Kingdom for an essay on "Three cases illustrating the causes of Dyspnœa."

### PUBLICATIONS RECEIVED.

From John Bale, Sons and Danielsson Ltd., London:

*Goitre.* By F. de Quervain. Translated by J. Snowman, M.D. 1924. Price 21s. net.
*Selections from the Works of Ambroise Paré.* Notes by Dorothea Waley Singer. 1924. Price 12s. 6d. net.

From The British Dental Association, London:

*The Preservation of the Teeth.* Recommendations of the British Dental Association Price 3d.

From The Cancer Research Fund (Ireland), Dublin:

*The Journal of Cancer.* No. 1. January, 1924.

From Cassell and Company Ltd., London:

*The Student's Handbook of Surgical Operations.* By Sir Frederick Treves, Bart., and Jonathan Hutchinson, F.R.C.S. Fourth Edition. 1924. Price 10s. 6d. net.

From J. and A. Churchill, London:

*Venereal Disease: its Prevention, Symptoms and Treatment.* By Hugh Wansey Bayly, M.C. Second Edition. 1924. Price 7s. 6d. net.

From William Heinemann, London:

*Insulin in General Practice.* By A. Clarke Begg, O.B.E., M.D., Ch.B. 1924. Price 5s. net.

From H. K. Lewis and Co., Ltd., London:

*Internal Derangements of the Knee-Joint.* By A. G. Timbrell Fisher, M.C., F.R.C.S. 1924. Price 12s. 6d. net.

From Humphrey Milford, London:

*Common Infections of the Female Urethra and Cervix.* By Frank Kidd, M.A., M.Ch. and A. Malcolm Simpson, B.A., M.B. [1924.] Price 7s. 6d. net.

From The School Medical Officer, Bristol:

*Annual Report of the School Medical Officer, City and County of Bristol Education Committee.* 1923. (Sixteenth Year.)

From Wakley and Son (1912) Ltd., London:

*Guy's Hospital Reports.* January, 1924. £2 2s. per annum.

From John Wright and Sons Ltd., Bristol:

*The Care and Cure of Cripple Children.* 1924. Price 2s. 6d.
General Index to Vols. I.—X. of *The British Journal of Surgery.* 1923. Price 10s. 6d. net. (To Subscribers 5s.)
*The Medical Annual,* 1924. Price 20s. net.
*A Whiff of Old Times: or One Hundred Extracts from Literature, for Medical Practitioners and others.* Collected by John Wishart, M.D., D.Sc., Ch.B. 1924. Price 3s.

Consistently prescribed by the British Medical profession for 40 years.

BENGER'S Food for INFANTS, INVALIDS & the AGED.

"Benger's Food has, by its excellence, established a reputation of its own."

"The British Medical Journal."

Sold in tins by all Chemists. Prices: 1/4; 2/3; 4/-; 8/6.

### E. & S. LIVINGSTONE,

MEDICAL PUBLISHERS,

### 16 & 17 Teviot Place, EDINBURGH.

LATEST PUBLICATIONS.

*Specialities:*

### THREE IMPORTANT WORKS IN PREPARATION.

Crown 8vo. 200 pp. Probable Price, **7s. 6d.** net.

### Clinical Studies in Epilepsy.

By **DONALD FRASER,** Consulting Physician, Royal Alexander Infirmary, Paisley, and Paisley District Mental Hospital, etc.

---

Crown 4to 200 pp. 261 Illustrations and Coloured Frontispiece. Probable Price, **21s.** net.

### Facial Surgery.

By **H. P. PICKERILL,** C.B.E., M.D., M.Ch., etc., Surgeon-in-Charge of Facial and Jaw Department, Dunedin Hospital, New Zealand, etc. With an Introduction by **Sir Wm. ARBUTHNOT LANE,** Bt., C.B., etc.

---

Demy 4to. 200 pp. With 400 half-tone Illustrations of X-ray Subjects and 100 Line Drawings. Probable Price, **21s.** net.

### An X-Ray Atlas of the Normal and Abnormal Structures of the Body.

With a Classification of Diseases, By **ARCHIBALD McKENDRICK,** F.R.C.S., D.P.H., Surgeon-in-Charge of the Surgical X-ray Department, Royal Infirmary, Edinburgh, and **CHARLES R. WHITTAKER,** F.R.C.S., F.R.S.E., assisted by others.

---

### JUST PUBLISHED.

Royal 8vo. 1,006+xx. pp. 474 Illustrations. **35s.** net. Postage **1s.**

### A Combined Text-Book of Obstetrics and Gynæcology.

By **J. M. MUNRO KERR,** M.D., F.R.F.P. & S. Glas., Professor of Obstetrics and Gynæcology, Glasgow University (Muirhead Chair); Obstetric Physician, Glasgow Royal Maternity and Women's Hospital; Gynæcological Surgeon, Glasgow Royal Infirmary, etc., etc.

**JAMES YOUNG,** D.S.O., M.D., F.R.C.S. Edin., Assistant Physician, Royal Maternity Hospital, Edinburgh; Assistant Gynæcologist, Royal Infirmary, Edinburgh; Lecturer in Clinical Gynæcology and Clinical Obstetrics, University of Edinburgh, etc.

**JAMES HAIG FERGUSON,** M.D., F.R.C.P. Edin., F.R.S.E., F.R.C.S., Gynæcologist, Royal Infirmary, Edinburgh; Obstetric Physician, Royal Maternity Hospital, Edinburgh; Lecturer in Clinical Gynæcology and in Clinical Obstetrics, University of Edinburgh, etc., etc.

**JAMES HENDRY,** M.A., B.Sc., M.B.Glas., Senior Assistant to the Muirhead Professor, University of Glasgow; Assistant Physician, Royal Maternity and Women's Hospital, etc.

---

Crown 8vo. 180 pp. 29 Illustrations. Price **6s.** net. Postage **5d.**

### The Insulin Treatment of Diabetes Mellitus.

By **P. J. CAMMIDGE,** M.D.Lond., D.P.H.Camb.

PUBLISHERS' NOTE.—Insulin is undoubtedly the greatest discovery yet made in connection with the treatment of Diabetes. This is the first book giving a concise summary of its properties, mode of action, and sphere of usefulness so far as at present known.

### E. & S. LIVINGSTONE'S MEDICAL PUBLICATIONS

*(continued).*

### RECENTLY PUBLISHED.

Demy 8vo Cloth. 278+vii. pp. **15s.** net.

Postage **8d.**

### Therapeutic Immunization in Asylum and General Practice.

By Wm. **FORD ROBERTSON,** M.D., late Pathologist to the Scottish Asylums.

"A book of absorbing scientific interest."

"The most up-to-date work on the subject."

Royal 8vo. 348+xx. pp. 66 Illustrations.

**21s.** net. Postage **1s.**

### The Mechanism of the Brain and the Function of the Frontal Lobes.

By Professor **LEONARDO BIANCHI,** of the Royal University of Naples. Authorised Translation from the Italian by **JAS. H. MACDONALD,** M.B., F.R.F.P.S. Glasgow; Mackintosh Lecturer in Psychological Medicine, University of Glasgow; Medical Superintendent, Govan District Asylum, Hawkhead, Glasgow. With a Foreword by Professor **C. LLOYD MORGAN.**

### HANDBOOKS in Preparation for Early Publication.

Seventh Edition. 630 pp. 34 Illustrations.
**12s. 6d.** net. Postage **6d.**

### Wheeler's Handbook of Medicine.

By **W. R. JACK,** M.D., F.R.F.P. & S.G., Physician, Glasgow Royal Infirmary; Lecturer, Clinical Medicine, University of Glasgow.

Second Edition. 260 pp. 46 Illustrations and 12 Coloured Plates. **10s. 6d.** net. Postage **6d.**

### Handbook of Skin Diseases.

By **FREDERICK GARDINER,** B.Sc., M.D., F.R.C.S.; Lecturer on Diseases of Skin, University, Edinburgh, etc.

Crown 8vo. 150 pp. Illustrated with 18 Full-page Plates. Probable Price, **6s.** net.

### An Outline of Endocrinology.

By **W. M. CROFTON,** B.A., M.D., Lecturer on Special Pathology, University College, Dublin; Pathologist Steeven's Hospital, Dublin.

### JUST PUBLISHED.

Crown 8vo. 592+xii. pp. 109 Illustrations.

**12s. 6d.** net. Postage **6d.**

### Handbook of Surgery.

By **GEO. L. CHIENE,** M.B., C.M., F.R.C.S. Edin., Surgeon, Royal Infirmary, Edinburgh; Senior Lecturer and Examiner, Clinical Surgery, University of Edinburgh; Formerly Assistant-Surgeon, Royal Hospital for Sick Children, Edinburgh. (Companion volume to Wheeler and Jack's "Medicine." Sixth Edition.)

Second Edition. 328+xvi. pp. 71 Illustrations.
8s. net. Postage 5d.

### Handbook of Anæsthetics.

By **J. STUART ROSS,** M.B., F.R.C.S.E., Lecturer in Practical Anæsthetics, University of Edinburgh; Instructor in Anæsthetics, Edinburgh Royal Infirmary. With chapters upon "Local and Spinal Anæsthesia," by W. Quarry Wood, M.D., F.R.C.S.E., Surg. Edin. Royal Infirmary; and upon "Intratracheal Anæsthesia," by W. Torrance Thomson, M.D., F.R.C.S.E, Anæsthetist to the Leith Hospital.

**A Full Catalogue of Messrs. Livingstone's Publications, including details of the well-known "CATECHISM SERIES," will be sent post free on application.**

